# A Rare Presentation of Intrasellar Mucocele Post-transsphenoidal Resection of a Non-secreting Pituitary Macroadenoma

**DOI:** 10.7759/cureus.44471

**Published:** 2023-08-31

**Authors:** Jessica Abou Chaaya, Mohamad Fleifel, Asma Arabi

**Affiliations:** 1 Internal Medicine, American University of Beirut Medical Center, Beirut, LBN

**Keywords:** pituitary gland, intrasellar mucocele, non-secreting pituitary macroadenoma, mucocele, pituitary adenoma

## Abstract

An enlarging sphenoid sinus mucocele can facilitate the growth of an intrasellar sinus mucocele. This subsequently leads to pituitary gland compression and endocrine abnormalities. We report the case of a 54-year-old man who underwent transsphenoidal resection of a non-secreting pituitary macroadenoma. Twenty years later he presented with headache, visual disturbances, erectile dysfunction, and poor libido and was diagnosed with a large sphenoid sinus mucocele that consequently extended into the sellar region. Based on the literature review, isolated intrasellar sinus mucocele post-transsphenoidal endoscopic surgery has been reported once. This is the first case of an intrasellar mucocele post-transsphenoidal resection to present with endocrine compromise on top of the compressive pituitary stalk symptoms.

## Introduction

Paranasal sinus mucocele is an encapsulated benign fluid-filled cavity. It can develop as a complication of chronic rhinosinusitis with destructive properties of the adjacent structures leading to compressive symptoms including visual disturbances and headache. Sphenoid sinus mucocele is usually rare and accounts for 1-2% of paranasal sinus mucoceles [1]. Intrasellar sinus mucocele can occur secondary to an enlarging sphenoid sinus mucocele leading to endocrine abnormalities secondary to compression on the pituitary gland. Isolated intrasellar sinus mucocele following transsphenoidal endoscopic surgery for resection of a pituitary macroadenoma was only reported once in the literature [2]. The underlying pathophysiology for such a phenomenon is still generally unknown to this date. We report here the case of a male patient with a history of transsphenoidal resection of a non-secreting pituitary macroadenoma causing pituitary stalk deviation, compressive symptoms, and hormonal abnormalities. Around 20 years later, he presented to our institution for headache, visual disturbances, and erectile dysfunction and was found to have an intrasellar mucocele. Based on our literature review, this will be the first reported case of an intrasellar mucocele associated with endocrine abnormalities while mimicking a recurrent non-secreting pituitary macroadenoma.

## Case presentation

Our patient is a 54-year-old man with a history of non-secreting pituitary macroadenoma. His history goes back to 20 years ago when the patient started to experience severe headaches associated with gradual vision loss in the left eye. There were no sexual compromises, breast pain, or galactorrhea. A 4×2×2 cm mass obliterating the sella turcica and eroding its floor was noted on an outside hospital magnetic resonance imaging (MRI). The mass occupied the right cavernous sinus, encasing the right internal carotid artery, and reached the medio-inferior aspect of the right cranial fossa. His prolactin (PRL) level was 585 µg/L (4.1-184) with a thyroid-stimulating hormone (TSH) of 1.12 uIU/mL (0.5 to 5.0) and a free thyroxine (FT4) level of 1.27 ng/dL (0.93-1.7) on diagnosis. The patient underwent an uncomplicated transsphenoidal resection of his pituitary lesion, with pathology showing benign pituitary macroadenoma. There was some residual left vision loss, but the headache ceased post-operation. A follow-up sella MRI done one month later revealed a residual tissue of the previously described macroadenoma and obliteration of the sphenoid sinus by a heterogeneous material that was attributed to the packing material. The patient underwent radiotherapy with a dose of 5040 Gy over 28 sessions for 38 days, and he was initiated on cabergoline 0.5 mg once weekly by his primary care physician. A sellar MRI 10 months later exhibited a residual tissue in the sella turcica characterized as hypointense on T1 weighted and heterogenous after gadolinium administration, and it measured approximately 3×2×1 cm.

**Figure 1:**
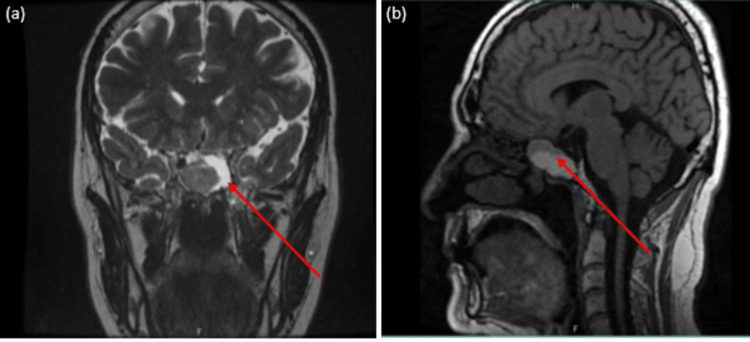
MRI sella with coronal (a) and (b) sagittal cuts showing the large sellar mass (red arrows)

**Figure 2:**
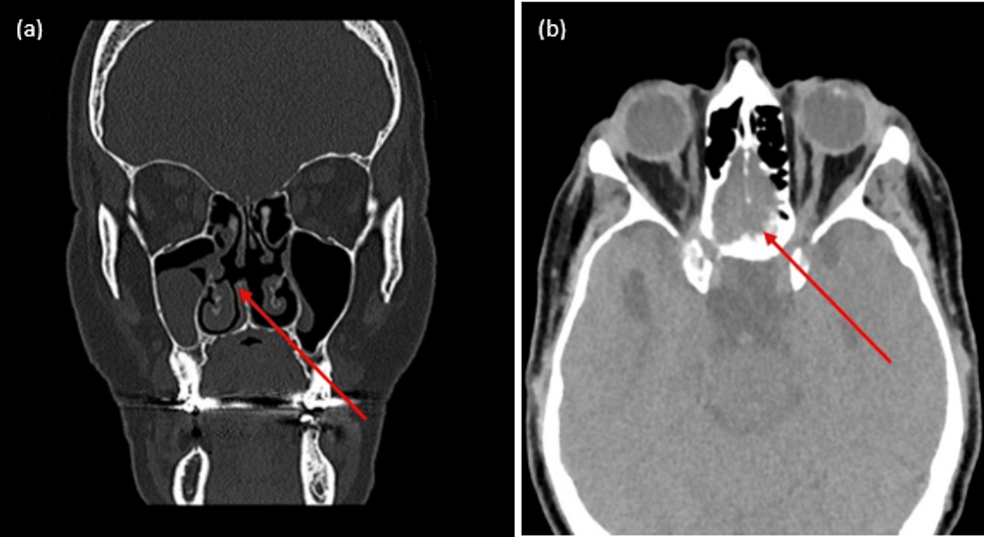
CT sinuses with coronal (a) and (b) axial cuts showing bilateral opacification (red arrows)

The patient was subsequently lost to follow-up until 20 years later when he presented to our institution for progressive headache and visual disturbances for the past few weeks. His headache was similar to the past one and his prior residual left vision loss was still present. However, new symptoms included acute right blurry vision, erectile dysfunction, and poor libido for the past week. The patient reported being on levothyroxine after getting diagnosed with hypothyroidism a few years prior to presentation. He was still on cabergoline. Paraclinical data included a random PRL of 1.99 µg/L, follicle-stimulating hormone (FSH) of 0.6 mIU/mL (1.5-12.4), luteinizing hormone (LH) less than 0.3 IU/L (1.7-8.6), FT4 of 1.09 ng/dL, random serum cortisol of 3.4 mcg/dL (2.47-11.9), insulin-like growth factor-1 (IGF-1) of 29.8 ng/mL (71.9-199), and adrenocorticotropic hormone (ACTH) of 28.3 pg/mL (10-60). An 8:00 AM serum cortisol was 4.91 mcg/dL (4.82-19.5). Sella MRI showed a large sellar mass measuring 4.2×2×2.2 cm compressing the optic chiasm on the left with a decrease in the caliber of the left optic nerve. The pituitary stalk deviated to the left with normal caliber (Figure 1). Pre-operative computed tomography (CT) of the sinuses described a complete opacification of the bilateral sphenoid sinus probably due to packing material (Figure 2). The packing and the postsurgical material from his first surgery in the sphenoid were inseparable from the sella, thus limiting its assessment. The patient was prepared for a second transsphenoidal resection and a bolus dose of 100 mg of hydrocortisone was given intravenously on-call to the operating room. Intra-operatively, the nasal septum was found to have a large perforation along with adhesions with the middle turbinate requiring excision. After penetrating the sphenoid ostium, a purulent clear secretion was noted and sent to pathology. Following adequate widening of the sphenoid ostium, the bulge of the sellar floor was identified and found to be pulsating and devoid of bone. The neurosurgery and otolaryngology teams decided not to breach the sellar floor and attributed the radiographic mass to an intrasellar mucocele. The pathology report indicated rare unremarkable respiratory epithelial cells, and cultures turned out to be negative.

Post-operatively, the patient was monitored for 24 hours in the hospital and was discharged later without any complications. Cabergoline and levothyroxine were stopped and the patient was put on prednisone 7.5 mg daily for seven days from discharge. The patient was placed on intramuscular (IM) injections of testosterone isocaproate 250 mg every four weeks, a follow-up clinic visit four months later showed major improvement in erections and libido. He did not have any fatigue or weakness. There was no documented hypotension. He was off prednisone and still off cabergoline. Paraclinical data, done in an outside hospital laboratory, exhibited a total testosterone level of 8.01 nmol/L (7.63-36.43), PRL of 20.3 µg/L, FT4 of 0.831 ng/dL (0.89-1.72), and 8:00 AM serum cortisol of 6.9 mcg/dL (3.7-19.4). The stimulated serum cortisol level (60 minutes after 250 mcg intravenous Synacthen) was 15.1 µg/dL (2.5-25). The follow-up MRI performed showed no evidence of residual mass. The patient continues to be followed up.

## Discussion

Paranasal sinus mucoceles are fluid-filled cavity of a benign nature and rarely occurs in the sphenoid sinus [3]. Sphenoid sinus mucoceles extending into the sellar area were reported in the literature in a few case reports [4,5}. As we previously mentioned and according to the literature review, isolated intrasellar sinus mucocele following a transsphenoidal endoscopic surgery for a pituitary macroadenoma was described once [2]. The beforementioned case and our patient both had previous transsphenoidal resection of pituitary macroadenoma several years prior to the development of mucocele. In the case reported by Tang et al, the 43-year-old male patient presented complaining of blurry vision with no other associated endocrine abnormalities 15 years post transsphenoidal resection for a non-secreting pituitary adenoma. MRI demonstrated a 4 cm lesion arising from the pituitary fossa and extending into the suprasellar region. In addition, there was a minimal enhancement of this structure after intravenous gadolinium, which favored a possible cystic degeneration within a pituitary macroadenoma. The exact pathophysiology is still unknown and Tang et al. explained that possible implantation of the mucosal sinus epithelium during the first surgical approach led to the later development of a mucocele isolated in the sellar region. The sellar region was devoid of mucus-secreting elements and the possible implanted epithelium would have become separated from the nasal chambers since the floor of the sellar was healed after his first operation. Their theory was supported by the imaging and operative findings of an intact sellar floor and empty sphenoid sinus [2]. In comparison, our patient possibly developed the mucocele from the sphenoid sinus extending into the sellar region. El Fiki et al. and Yokoyama et al. each reported three cases of sphenoethmoidal mucoceles sinus extending into the sellar and suprasellar areas, but none of these were preceded by an intervention such a transsphenoidal surgery [4,5]. On the other hand, this rare complication has been reported in transsphenoidal operations for pituitary adenomas and according to Moriyama et al., the mucocele of the sphenoid sinus usually develops 15 to 25 years after the initial surgical operation for sinusitis but never led to a clinical presentation like our patient (i.e., low libido and erectile dysfunction in addition to the other hormonal imbalances) and was only confined to headache and visual disturbances [6].

Our patient had several endocrine abnormalities in comparison to the previously mentioned reported patients. First, the patient had hyperprolactinemia and was maintained on cabergoline, which can be explained by a pituitary stalk effect rather than a secreting mass since his first and second pathology reports were both nonrevealing of any lactotroph cells suggestive of a prolactinoma. Second, he was complaining of a long-standing erectile dysfunction compatible with his low FSH and LH levels, hypothyroidism, and low cortisol levels. These abnormalities can be attributed to the large mucocele compromising the hormonal axes of the pituitary gland and also likely due to previous surgery and radiotherapy. Furthermore, he exhibited symptoms of compressive symptoms manifesting as the acute onset of blurry vision in his unaffected right eye and severe headache. The endocrine disturbances, the imaging findings, and the operative discoveries suggest that the patient developed a mucocele as a complication of his first transsphenoidal resection, enlarging through time leading to significant compression and subsequent clinical symptoms. The previously reported cases explained the occurrence of mucocele as a potential complication of a transsphenoidal surgery leading to compressive symptoms such as headache or blurry vision, but none had the same endocrinological abnormalities mimicking a recurrent pituitary macroadenoma as in our patient. Gozgec E. et al. presented an unusual case of hypopituitarism secondary to an invading sphenoid mucocele into the sellar area as an initial presentation and not secondary to a surgical intervention [7].

## Conclusions

Mucoceles are mucus-rich cystic lesions walled by the epithelium, and they are relatively rare to be found in the intrasellar region. Although histologically benign, such enlarging mucoceles can possess a destructive behavior that mimics a pituitary macroadenoma leading to neuroophthalmic manifestations, and in our case, endocrinologic compromises. The correct diagnosis and adequate follow-up are definitely essential to minimize long-term damage.
